# USING A STRESS PROCESS MODEL TO EXAMINE RACIAL DIFFERENCES IN CAREGIVER WELL-BEING AND HEALTH

**DOI:** 10.1093/geroni/igz038.1807

**Published:** 2019-11-08

**Authors:** Adrian Badana, William E Haley

**Affiliations:** 1 School of Aging Studies, University of South Florida, Tampa, Florida, United States; 2 University of South Florida, Tampa, Florida, United States

## Abstract

Current research must utilize nationally-representative samples of older adults and their family caregivers to accurately reflect the growing diversity of the United States. This study aims to use a stress process model to examine potential racial differences in caregiving in a population-based sample of 844 White and 389 Black family caregivers in the United States. We conducted 3 x 2 x 2 (relationship type x race x dementia care status) factorial ANOVAs to examine potential differences in caregiving stressors, appraisals, resources, and mental and physical health outcomes among primary family caregivers. Results indicated significant racial differences in caregiving on several stress process measures. Although Black caregivers reported more caregiving stressors, compared to White caregivers, they tended to report more positive appraisals of caregiving and more caregiving resources. Dementia caregivers tended to report greater caregiving stressors and worse measures of appraisal compared to non-dementia caregivers. There was a significant two-way interaction among relationship type and dementia care status for the caregiving stressor, hours of care. A stress process model can allow researchers to investigate various factors associated with racial differences in caregiving.

